# Phytochemical screening, antiobesity, antidiabetic and antimicrobial assessments of *Orobanche aegyptiaca* from Palestine

**DOI:** 10.1186/s12906-021-03431-x

**Published:** 2021-10-08

**Authors:** Nidal Jaradat, Mohammad Qadi, Iyad Ali, Fatima Hussein, Linda Issa, Doaa Rashdan, Manal Jamoos, Re’as Najem, Abdulraziq Zarour, Mohammad Arar

**Affiliations:** 1 Department of Pharmacy, Faculty of Medicine and Health Sciences, An-Najah National University, Nablus, P.O. Box. 7, Palestine; 2 Department of Biomedical Sciences, Faculty of Medicine and Health Sciences, An-Najah National University, Nablus, P.O. Box. 7, Palestine

**Keywords:** *Orobanche aegyptiaca*, Qualitative phytochemistry, Antilipase, Anti-α-amylase, Antimicrobial

## Abstract

**Background:**

Microbial resistance, diabetes mellitus, and obesity are global health care problems that have posed a serious threat to both human and environmental ecosystems. The goals of the present investigations are to investigate the phytoconstituents, antilipase, anti-α-amylase, and antimicrobial activity of *Orobanche aegyptiaca* Pers. (OA) from Palestine.

**Methods:**

Identification of the phytoconstituents of OA plant petroleum ether, methylene chloride, chloroform, acetone, and methanol extracts were conducted using pharmacopeia’s methods, while porcine pancreatic lipase and α–amylase inhibitory activities were examined using p-nitrophenyl butyrate and 3,5-dinitro salicylic acid methods, respectively. Moreover, the antimicrobial activity was evaluated utilizing broth microdilution assay against eight bacterial and fungal strains.

**Results:**

The phytochemical screening results showed that the methanol extract of the OA plant is rich in phytochemical components, also this extract has powerful antilipase potential with an IC_50_ value of 19.49 ± 0.16 μg/ml comparing with the positive control (Orlistat) which has antilipase activity with IC_50_ value of 12.3 ± 0.35 μg/ml. Moreover, the methanol and chloroform extracts have powerful α-amylase inhibitory activity with IC_50_ values of 28.18 ± 0.22 and 28.18 ± 1.22 μg/ml, respectively comparing with Acarbose which has α-amylase inhibitory activity with IC_50_ dose of 26.3.18 ± 0.28 μg/ml. The antibacterial results showed that the methylene chloride extract exhibited the highest antibacterial activity among the other OA plant extracts with a MIC value of 0.78 mg/ml against *S. aureus,* while, the methylene chloride, petroleum ether, and chloroform extracts of the OA plant showed potential antifungal activity against *C. albicans* strains with MIC value of 0.78 mg/ml.

**Conclusion:**

The OA methanol and chloroform extracts could be excellent candidates as antilipase and anti-α-amylase bioactive materials. In addition, methylene chloride, petroleum ether, and chloroform extracts could be potential natural antimicrobial products.

## Introduction

Phytotherapy is a famous ancient type of therapeutics that arises from plants that positively affect the human body for various diseases. Not so long time ago, phytotherapy was the only medicine, which then gradually spread in all developed and developing countries [1]. However, several studies from different global regions recorded the increase in the use of plants to cure various human diseases [2].

There has been widespread interest in obesity globally in the past few years, and its implications on public health, and its correlation with serious non-communicable diseases, some of which are major causes of death worldwide, such as diabetes mellitus, cardiovascular diseases, musculoskeletal disorders, and several kinds of cancer including liver, gallbladder, kidney, colon, prostate, and breast cancer [3, 4].

In 2016, the statistics showed that more than1.9 a billion adults aged 18 years and over were overweight. Also, it found that over 650 million of them were obese. Moreover, about 39% of the world’s adult population, 39% of men, and 40% of women were overweight; besides, about 13% of world adults, 11% of men, and 15% of women were obese. On the individual level, people can reduce energy intake from fats and sugars, increase the consumption of fruit and vegetables, and do more regular physical activities [5].

Around the world, diabetes disease has been rising rapidly and causing several complications including; lower limb amputation, stroke, heart attacks, blindness, and kidney failure, thus increasing morbidity. The number of people with diabetes has increased from 108 million in 1980 to 422 million in 2014. In 2016, an estimated 1.6 million deaths were directly caused by diabetes. Another 2.2 million deaths were related to high blood glucose in 2012; it is also expected to increase in the coming years [5].

Recently, antimicrobial resistance became a huge global problem and especially for WHO. This problem is spreading quickly, which causes many dangerous complications in diseases and, as a result, an increase in the percentage of mortality and morbidity everywhere, especially in hospitals. Antibiotic resistance occurs when bacteria change in response to the great use of these medications, commonly because of misuse of antibiotics. About 11% of patients who undergo surgery are infected in the process. In 2016, about half a million people had multi-drug-resistant tuberculosis globally. Drug resistance is starting to complicate the combat against HIV and malaria too. Patients with MRSA (Methicillin-Resistant *Staphylococcus aureus*) are considered to be 64% more likely to die than people with a non-resistant form of the infection [6].

*Orobanche aegyptiaca* Pers. (Orobanchaceae), commonly known as Egyptian broomrape, is a perennial herbaceous plant with purple flowers and tiny leaves. It grows in grassland areas such as the Middle East and more overproduces via seeds [7]. In folk medicine, it is used internally to treat infectious diseases and externally to assess the wound healing process, pores occlusive, and psoriasis treatment [8].

This study aims to find out the antimicrobial effects of five extracts of *O. aegyptiaca* on seven different types of bacteria, and one fungus as well as their effect on specific body enzymes, mainly lipase, and amylase, to see if this plant can be a potential source of the lead metabolites for the treatment of diabetes, obesity, excess weight, high cholesterol, triglyceride indexes, and other related diseases.

## Materials and methods

### Sample’s collection and preparation

The OA plant aerial parts (leaves and stems) were collected from the Nablus region of the West Bank area, Palestine, in August 2019. The OA plant was identified in the Herbal Products Laboratory, Department of Pharmacy at An-Najah National University by the pharmacognosist Dr. Nidal Jaradat, and the voucher specimen was deposited in the Herbal Products Laboratory within a code of Pharm-PCT-1746. The collected samples were cut into small parts, rinsed several times with distilled water, and identified in the Pharmacognosy Laboratory at An-Najah National University. It was then air-dried for 7 days. The dried OA samples were ground mechanically (Moulinex model, Uno, China) into a fine powder to facilitate extraction and kept in paper bags for further solvent extraction processes.

### Extraction of the phytochemical constituents

Twenty grams of the powdered OA sample was extracted successively by a soxhlet extractor, according to the method adopted by Abdel-Aal et al. by utilizing different organic solvents with analytical reagent quality (Sigma Aldrich, Germany). These solvents were petroleum ether (40–60 °C), methylene chloride (39.6 °C), chloroform (61.15 °C), acetone (56 °C), and finally methanol (64.7 °C). To ensure the complete extraction process, exhaustive extraction was applied with each solvent for 10 h. Extracts of different organic solvents were collected separately into dry clean beakers, after that, they were recovered from the solvents by evaporation in a rotary evaporator (Heidolph, OB2000, Germany) at 40 °C, then the samples were dried in desiccators for 1 h and the obtained extracts were weighed and the percentage of each extract was determined [9].

### Phytochemical analysis

The phytochemical screening tests were conducted according to standard analytical methods to identify the presence of primary and secondary metabolic groups including carbohydrates, flavonoids, saponins, glycosides, alkaloids, tannins and phenols, protein, and terpenoids [10].

### Pancreatic lipase inhibition method

The porcine pancreatic lipase inhibitory test was conducted according to the biochemical method adapted by Jaradat et al. [11]. One mg/ml of OA plant extract stock solution was dissolved in 10% dimethyl sulfoxide (DMSO) (Riedeldehan, Germany), from which five different solutions were prepared with various concentrations: 50, 100, 200, 300, and 400 μg/ml. One mg/ml stock solution of pancreatic lipase (Sigma, St. Louis, MO, USA)) was produced immediately before being used. A stock solution of PNPB (p-nitrophenyl butyrate) (Sigma-Aldrich, Schnelldorf, Germany) was prepared by dissolving 20.9 mg of PNPB in 2 ml of acetonitrile. 0.1 ml of porcine pancreatic lipase (1 mg/ml) was added to test tubes containing 0.2 ml of the previously prepared concentrations of plant extract. The resulting mixtures were then made up to 1 ml by adding Tri-HCl (Sigma-Aldrich, Schnelldorf, Germany) solution (pH 7.4) and incubated (Nuve, Turkey) at 37 °C for 15 min. After the incubation period, 0.1 ml of PNPB solution was then added to each test tube. The mixture was again incubated for 30 min at 37 °C. Pancreatic lipase activity was determined by measuring the hydrolysis of p-nitrophenyl butyrate to p-nitrophenol at 405 nm using a UV-visible spectrophotometer (Jenway 7135, England). The same procedure was repeated for the aqueous and organic extracts and for Orlistat (Sigma-Aldrich, Schnelldorf, Germany) which was used as a positive control utilizing the same concentrations as mentioned above. The established tests were performed in triplicates. However, the lipase enzyme inhibitory potential was measured utilizing the next equation:

$$\mathrm{I}\ \left(\%\right)\kern0.5em =\kern0.5em \left[\mathrm{ABSblank}\kern0.5em \hbox{-} \kern0.5em \mathrm{ABStest}\right]/\left[\mathrm{ABSblank}\right]\Big)\ast 100\%$$

### α-Amylase inhibitory method

The α-amylase inhibitory potential was done as described before [12], with some modifications. Briefly, a plant working solution (1 mg/ml) was prepared by mixing 25 mg of each plant extract in 10% of DMSO. This solution was then diluted by the buffer to obtain different dilutions (10, 50, 70, 100, 500 μg/ml). Later on, an α-amylases enzyme stock solution (2 units/ml) was prepared by dissolving 12.5 mg of α-amylases enzyme (Sigma, Mumbai, India) powder in a minimum amount of 10% DMSO, and buffer solution was added up to 100 ml. Then a corn starch solution was prepared by dissolving 1 g of starch in 100 ml distilled water. A 200 μl from each plant extract stock solution was mixed with 200 μl of α-amylase stock solution and incubated for 10 min at 30 °C in a water bath. After that, 200 μl of corn starch solution was added and incubated for 3 min at 30 °C. Moreover, 3,5-dinitro salicylic acid (Sigma, Mumbai, India) was added and boiled in a water bath at 85–90 °C for 10 min and after the solution has cooled, 5 ml of distilled water and prepared blank solutions were added throughout the replacement of the OA extracts with 200 μl of buffer solution. In addition, the commercial anti-amylase drug Acarbose (Sigma, Mumbai, India) was utilized as a positive control. The optical activity of the prepared solutions was assessed at 540 nm utilizing a UV-Visible spectrophotometer. The established tests were conducted in triplicates.

The α-amylase inhibitory potential was calculated utilizing the following equation:

$$\mathrm{I}\ \left(\%\right)\kern0.5em =\kern0.5em \left[\mathrm{ABSblank}\kern0.5em \hbox{-} \kern0.5em \mathrm{ABStest}\right]/\left[\mathrm{ABSblank}\right]\Big)\ast 100\%$$

Where I (%), is the α-amylase inhibitory percentage.

### Antimicrobial

#### Microbial isolates

The examined bacterial and fungal isolates were obtained from American Type Culture Collection (ATCC), in addition to a clinically confirmed Methicillin-Resistant *Staphylococcus aureus* (MRSA). The selected species of microorganisms are frequently isolated at clinical settings in our region and some possess multidrug resistance. The isolates included three Gram-positive species: *Staphylococcus aureus* (ATCC 25923), MRSA a clinical strain, and *Enterococcus faecium* (ATCC 700221) and four Gram-negative species: *Klebsiella pneumoniae* (ATCC 13883) *Proteus vulgaris* (ATCC 8427), *Escherichia coli* (ATCC 25922) and *Pseudomonas aeruginosa* (ATCC 9027). Meanwhile, the fungal isolate is *Candida albicans* (ATCC 90028).

#### Antimicrobial test

The antimicrobial activity of OA plant five extracts was conducted according to National Committee for Clinical Laboratory Standards (NCCLS) guidelines utilizing the broth microdilution assay [13]. Each extract of the OA plant was dissolved to a concentration of 100 mg/ml After that, each produced solution was serially micro-diluted (2 folds) 10 times in sterile Mueller-Hinton broth (Himedia, India). The dilution processes were performed under aseptic conditions in 96 well plates (micro broth plate (Greiner bio-one, North America). In the micro-wells that were assigned to evaluate the antibacterial activity of each extract, micro-well number eleven contained extract-free Mueller-Hinton broth, which was used as a positive control for microbial growth. On the other hand, micro-well number twelve contained extract-free and microbial-free Mueller-Hinton broth, this well was used as a negative control for microbial growth. Micro-wells numbers (1–11) were inoculated aseptically with the test microbes that were previously prepared in Mueller-Hinton broth to obtain a standard bacterial concentration based on clinical and laboratory standards institute. For each plant extract tested, another control was added which consisted of a plant extract alone serially diluted to obtain the same concentrations of extract tested for antimicrobial activity, this control was run to be sure there is no contamination and turbidity, and to make sure changes in color are not due to oil itself. Each extract of OA plant antimicrobial activity was carried out in triplicates. All the inoculated plates were incubated at 35 °C. Regarding the *C. albicans,* the same method was used but using RPMI media instead of Mueller-Hinton broth. The incubation period lasted for about 18–24 h for those plates inoculated with the test bacterial strains and for about 48 h for those plates inoculated with *C. albicans*. The lowest concentration of each extract of OA plant at which no visible microbial growth in that micro-well was observed, was considered as the minimal inhibitory concentration (MIC) of the examined OA plant extract. The antimicrobial activity was evaluated using known antimicrobial agents namely Ampicillin and Ciprofloxacin which were used as positive controls for antibacterial activity and Fluconazole was used as a positive control for antifungal activity.

#### Statistical assessment

Each established experiment in our study was performed in triplicate (*n* = 3). The findings of the five OA plant extracts were presented as means with standard deviations (SD). A t-test was used to compare averaged data. When the *p*-value was less than 0.05, statistical significance was recognized and documented.

## Results and discussion

### Phytochemical screening

The preliminary phytochemical screening tests revealed the presence of terpenoids and tannins in OA petroleum ether extract while reducing sugars and terpenoids were found in the methylene chloride extract. In addition, terpenoids were the only phytochemical class identified in the OA) acetone extract. However, the methanolic OA extract was enriched with multiple phytochemical classes including alkaloids, reducing sugars, glycosides, phenol, terpenoids, tannins, flavonoids, proteins, and amino acids as presents in Table 1.

**Table 1:** Phytochemical screening test of OA petroleum ether, methylene chloride, chloroform, acetone, and methanol extracts

Phytochemical classes	Petroleum ether	Methylene chloride	Chloroform	Acetone	Methanol
Alkaloids	Negative	Negative	Negative	Negative	Positive
Carbohydrates	Negative	Negative	Negative	Negative	Negative
Reducing Sugars	Negative	Positive	Negative	Negative	Positive
Glycosides	Negative	Negative	Negative	Negative	Positive
Saponins	Negative	Negative	Negative	Negative	Negative
Phenol	Negative	Negative	Negative	Negative	Positive
Terpenoids	Positive	Positive	Positive	Positive	Positive
Tannins	Positive	Negative	Negative	Negative	Positive
Flavonoids	Negative	Negative	Negative	Negative	Positive
Proteins and Amino Acids	Negative	Negative	Negative	Negative	Positive

### Porcine pancreatic enzyme inhibitory activity

Obesity is a serious metabolic disorder caused by an imbalance between energy intake and expenditure. It is a major risk factor for cardiovascular, metabolic, endocrine, and cancer illnesses [14]. Accordingly, the use of medicinal plants would be a great and safe medicinal alternative in the treatment of obesity. They have been used to promote beneficial health effects, especially for the prevention of pathophysiological conditions such as obesity, dyslipidemia, diabetes, hypertension, and cancer.

The lipolytic pancreatic lipase enzyme is synthesized and secreted by the pancreas, plays a key role in the efficient digestion of lipids, and is responsible for the hydrolysis of 50–70% of total dietary lipids. The anti-lipase effect is one of the most widely studied mechanisms in determining the potential efficacy of natural products as anti-obesity agents [15].

In the last two decades, global interest has been focused on the effects of plants, especially those that classified as folkloric medicinal ones for the treatment of obesity and for controlling excess weight because these plants have been utilized from ancient times, and their toxic and other side effects have been observed and documented if found [16, 17].

**Fig. 1:**
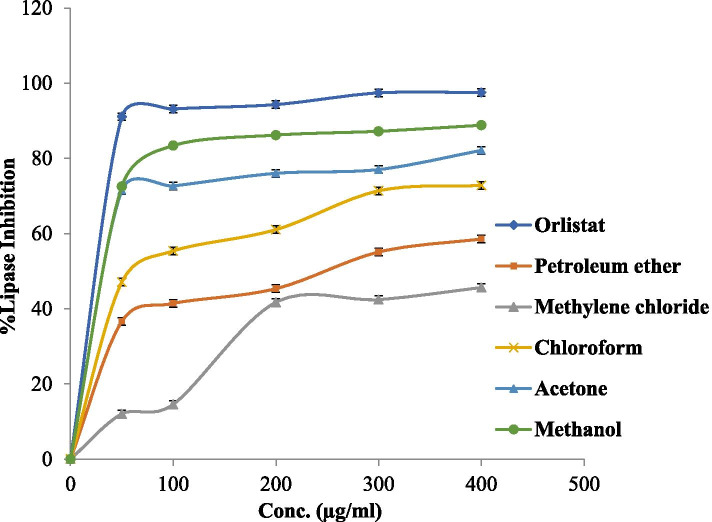
Lipase inhibitory activity values of the OA five extracts and Orlistat

The porcine pancreatic enzyme inhibitory characters of the five OA plant extracts and the positive control drug (Orlistat) were noticed according to a dose-dependent manner. Various solutions of the Orlistat and OA plant extracts were prepared in escalating doses as shown in Table 2. The IC_50_ values for the drug and plant extracts were calculated and the degree of lipase inhibition was plotted as shown in Fig. 1. The results revealed that the methanol OA extract has powerful anti-lipase potential followed by acetone, chloroform, and petroleum ether, with IC_50_ values of 19.49 ± 0.16, 26.3 ± 0.23, 63.09 ± 0.2 and 199.5 ± 0.2 μg/ml, respectively, comparing with the positive control (Orlistat) which has antilipase activity with an IC_50_ value of 12.3 ± 0.35 μg/ml. However, the anti-lipase IC_50_ value of the OA methylene chloride extract was more than 1000 μg/ml (inactive).

**Table 2:** The MIC values (mg/ml) of (OA) plant five extracts

Extracts	Bacterial isolates							Fungal isolate
	Gram-Positive			Gram-negative				
	MRSA	***S. aureus***	***E. faecium***	***E. coli***	***K. pneumoniae***	***P. vulgaris***	***P. aeruginosa***	***C. albicans***
Acetone	3.125	1.56	1.56	3.125	3.125	3.125	1.56	1.56
Methanol	6.25	6.25	6.25	R	R	R	R	R
Methylene chloride	1.56	0.78	1.56	3.125	3.125	3.125	3.125	0.78
Petroleum ether	1.56	1.56	1.56	6.25	6.25	6.25	3.125	0.78
Chloroform	1.56	1.56	1.56	1.56	1.56	1.56	3.125	0.78

### The α-amylase inhibitory activity

The in vitro anti-diabetic activity of the five traditional (OA) medicinal Palestinian plant extracts was investigated by the assessment of their α-amylase inhibitory effect. Acarbose was used as a positive control to compare the (OA) plant α-amylase inhibitory activity.

However, all the studied α-amylase inhibitory assays in the five extracts including Acarbose were conducted at various concentrations.

**Fig. 2:**
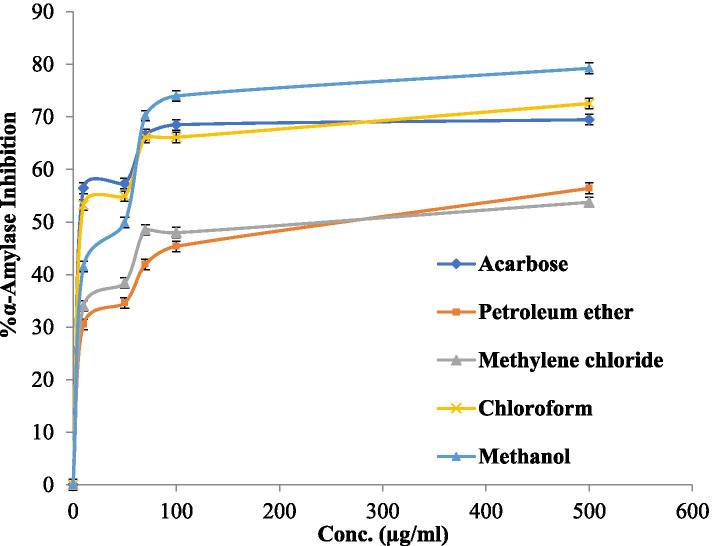
α-Amylase inhibitory activity values of OA five extracts and Acarbose

The α-amylase inhibitory activity of the commercial antidiabetic drug Acarbose and OA five extracts are presented in Fig. 2. The results revealed the methanol and chloroform extracts of the OA plant have powerful α-amylase inhibitory activity with IC_50_ values of 28.18 ± 0.22 and 28.18 ± 1.22 μg/ml followed by methylene chloride and petroleum ether which have anti-α-amylase activity IC_50_ doses of 151.3 ± 0.13 and 190.54 ± 0.27, respectively. Acarbose which was used as a positive control has an IC_50_ dose of 26.3.18 ± 0.28 μg/ml. While the α-amylase inhibitory activity of OA acetone extract was more than 1000 μg/ml (inactive).

The (OA) methanolic extract showed strong inhibitory activity against lipase and α-amylase metabolic enzymes. This means that this extract can break down the digestion of complex carbohydrates and fats within the gut which can directly affect the energy uptake and storage causing the loss of weight [18].

### Antimicrobial activity

The in vitro antibacterial activity of five OA plant extracts against bacterial strains (both Gram-positive and Gram-negative bacteria) in addition to one fungal strain was assayed using broth microdilution assay. Table 2 revealed that the acetone extract of the OA plant has the highest antibacterial inhibitory activity against *S. aureus, E. faecium,* and *P. aeruginosa* with MIC doses of 1.56 mg/ml. While, the methanol extract inhibited only the growth of *S. aureus, E. faecium,* and MRSA (Gram-positive bacteria) with MIC values of 6.25 mg/ml. Moreover, the methylene chloride extract revealed the highest antibacterial activity among other (OA) plant extracts with a MIC value of 0.78 mg/ml against *S. aureus,* followed by *E. faecium* and MRSA with MIC values of 1.56 mg/ml. In addition, the petroleum ether extract has potential antibacterial activity against *S. aureus, E. faecium,* and MRSA with a MIC dose of 1.56 mg/ml. Finally, the chloroform extract has the highest inhibitory activity against *S. aureus, E. coli, K. pneumoniae, P. vulgaris, E. faecium,* and MRSA with a MIC value of 1.56 mg/ml. Briefly, against the growth of *S. aureus* strain, the methylene chloride extract showed the best activity with a MIC value of 0.78 mg/ml and against *E. coli, K. pneumoniae,* and *P. vulgaris the* chloroform extract revealed the best inhibitory activity. While, against *E. faecium* growth, the acetone*,* methylene chloride, petroleum ether, and chloroform extracts of the OA plant have the same MIC value (1.56 mg/ml). Moreover, against *P. aeruginosa,* the Acetone extract showed the highest inhibitory activity with a MIC value of 1.56 mg/ml. In addition, against MRSA, methylene chloride, petroleum ether, and chloroform revealed the highest inhibitory activity.

In fact, the methylene chloride, petroleum ether, and chloroform extracts of the OA plant showed potential antifungal activity against *C. albicans* strains with a MIC value of 0.78 mg/ml. All in all, it is observed that the greatest inhibition against Gram-positive bacterial growth was caused by methylene chloride extract and the greatest inhibition against Gram-negative bacterial growth was caused by chloroform extract while the greatest inhibition against fungal growth was caused by methylene chloride, petroleum ether, and chloroform OA extracts.

A study conducted by Genovese et al. found that *O. crenata* leaves acetone extract has antimicrobial activity against *S. aureus, E. faecium, E. coli, K. Pneumonia, P. aeruginosa,* and *C. albicans* with MIC value of 752, 1505, 6023, 6023, 3011, and 188 μg/ml, respectively [19].

The current study phytochemical screening results revealed that the methanolic extract is the only one that contains phenolic components including flavonoids, tannins, and simple phenols. Actually, various studies have shown that there is a positive correlation between the presence of tannin, flavonoid, and simple phenol compounds and inhibitory potentials on pancreatic lipase and α-amylase. Also, high levels of polyphenolic compounds have been shown to reduce the potency of α-amylase and lipase by either inhibiting or interacting with a specific component of these enzymes [20–22]. According to Agrios [23], plants with high polyphenolic content have the potentials for enzyme inhibitory capacity. The current study outcomes are in agreement with a study of Gironés-Vilaplana et al. [24] which noticed a strong positive relationship between the lipase inhibitory activity and phenolic content in many fruits from Latin America. On the other side, Boath et al. [25] revealed that herbal polyphenols are not necessary for lipase inhibition [26]. Besides, methanolic OA extract was the only one that contained alkaloid compounds. However, Salih et al. investigated alkaloid content in OA utilizing High-Performance Liquid Chromatography (HPLC) and its antioxidant activity. Four alkaloids were identified in OA extract including oxosparteine, isosparteine, spartein, and lupanine), while the inhibition rate of free radicals was 85.31% [27].

In fact, methanolic OA extract contains various types of phenolic components and alkaloids which exhibited antioxidant potential, as it is well established that these kinds of phytochemical classes have strong antioxidant potential. Various studies have verified the correlation between antioxidant activity, antilipase, and anti-α-amylase effects [28–30]. Moreover, the presence of terpenoids in all the screened OA plant extracts provided them with broad-spectrum antimicrobial activity [31]. Also, the petroleum ether, methylene chloride, chloroform, acetone, and methanol AO extracts contained terpenoids and inhibited most of the tested microbial species.

To the best of the authors’ knowledge, no previous studies were conducted on (OA) plant antilipase, anti-amylase, and antimicrobial activities.

Further isolation and identification of the active constituents that are responsible for the antilipase, anti-amylase, and antimicrobial activities, in addition to in-vivo studies are required to approve these biological activities of the OA plant.

## Conclusions

The results revealed that the methanol OA extract has powerful antilipase potential compared with the positive control (Orlistat) and the methanol and chloroform plant extracts have powerful α-amylase inhibitory activity compared with Acarbose. Moreover, the antimicrobial assay results revealed that the methylene chloride OA extract has the greatest inhibitory activity against bacterial growth, while the greatest inhibition against fungal growth was noticed by methylene chloride, petroleum ether, and chloroform OA extracts. The present work attempts to expand the applications of herbal medicine in the drug discovery system for the manufacturing of medicines from nature.

## Data Availability

The data used to support the findings of this study are included in the article.
